# A multi-variable predictive warning model for cervical cancer using clinical and SNPs data

**DOI:** 10.3389/fmed.2024.1294230

**Published:** 2024-02-22

**Authors:** Xiangqin Li, Ruoqi Ning, Bing Xiao, Silu Meng, Haiying Sun, Xinran Fan, Shuang Li

**Affiliations:** ^1^Department of Obstetrics and Gynecology, Tongji Hospital, Tongji Medical College, Huazhong University of Science and Technology, Wuhan, China; ^2^Cancer Biology Research Center, Tongji Hospital, Tongji Medical College, Huazhong University of Science and Technology, Wuhan, China

**Keywords:** cervical cancer, predictive model, germline mutation, SNPs, clinical features

## Abstract

**Introduction:**

Cervical cancer is the fourth most common cancer among female worldwide. Early detection and intervention are essential. This study aims to construct an early predictive warning model for cervical cancer and precancerous lesions utilizing clinical data and simple nucleotide polymorphisms (SNPs).

**Methods:**

Clinical data and germline SNPs were collected from 472 participants. Univariate logistic regression, least absolute shrinkage selection operator (LASSO), and stepwise regression were performed to screen variables. Logistic regression (LR), support vector machine (SVM), random forest (RF), decision tree (DT), extreme gradient boosting(XGBoost) and neural network(NN) were applied to establish models. The receiver operating characteristic (ROC) curve was used to compare the models’ efficiencies. The performance of models was validated using decision curve analysis (DCA).

**Results:**

The LR model, which included 6 SNPs and 2 clinical variables as independent risk factors for cervical carcinogenesis, was ultimately chosen as the most optimal model. The DCA showed that the LR model had a good clinical application.

**Discussion:**

The predictive model effectively foresees cervical cancer risk using clinical and SNP data, aiding in planning timely interventions. It provides a transparent tool for refining clinical decisions in cervical cancer management.

## 1 Introduction

In terms of incidence (6.5%) and mortality (7.7%), cervical cancer is the fourth most common cancer in women worldwide (1). The incidence (5.2%) and mortality (5.3%) of cervical cancer in China are much higher than in developed countries (2). Cervical cancer develops due to a complicated interaction between elements influencing the virus’s carcinogenic potential and host characteristics associated with susceptibility to chronic infection and tumor formation (3). Although persistent high-risk types of human papillomavirus (hrHPV) infection play a critical role in the development of cervical cancer, this alone cannot explain the malignancy (4). In addition to factors such as high-risk sex, sexually transmitted diseases, preterm births, multiple births, use of oral hormonal contraceptives and smoking that may affect human papillomavirus (HPV) infection (5, 6), numerous studies have demonstrated the association between simple nucleotide polymorphisms (SNPs) as genetic factor, which may have an impact on gene expression or protein function, and cervical carcinogenesis. The effectiveness of the immune response to HPV antigens may be altered by genetic variations in human leukocyte antigen (HLA) molecules, which may retard the progression of cervical cancer (7). For instance, the HLA class II DRB1*1302 allele protects against the advancement of low-grade squamous intraepithelial lesion (LSIL) into grade 3 cervical intraepithelial neoplasia (CIN3) (8). Furthermore, cervical cancer has been associated with specific genetic SNPs crucial for DNA repair, apoptosis, and cell metabolism (9–11).

In addition to health education and HPV vaccine acting as primary prevention measures, the clinical screening and diagnosis of cervical cancer is primarily based on the three-step procedure (hrHPV test, Papanicolaou test, and colposcopy). Regular hrHPV test and Papanicolaou test are recommended for early cervical lesion detection of at-risk populations, and those with abnormal test results were referred to colposcopy to receive timely and reasonable treatment. In order to increase the risk awareness of individuals in the preclinical stage of cervical cancer, we developed a predictive warning model for clinical diagnosis of cervical cancer and precancerous lesions combining clinical and mutational features. We subsequently validated and evaluated these models, considering them as potential risk indicators and supplementary diagnostic tools. This approach may contribute to the development of a cost-effective screening test for early cervical cancer detection, ultimately benefiting public health.

## 2 Materials and methods

### 2.1 Study population

The 474 subjects were recruited from the cervical specialist outpatient and inpatient departments of the Department of Obstetrics and Gynecology, Tongji Hospital, Tongji Medical College, Huazhong University of Science and Technology. There were 211 subjects in the patient group and 263 subjects in the control group. Inclusion criteria for the patient group were as follows: (i) age range of 18–75 years, (ii) Han Chinese ethnicity, (iii) no prior surgery, radiotherapy, chemotherapy, or other related treatment for cervical cancer or precancerous lesions, (iv) no previous personal history of other tumors, (v) pathology confirmed as CIN2, CIN3 or squamous carcinoma. All subjects in the patient group had their postoperative pathological results tracked, and the highest grade lesion was utilized as the final diagnosis. Inclusion criteria for the control group were as follows: (i) age range of 18–75 years, (ii) Han Chinese ethnicity, (iii) no prior history of cervical precancerous lesions or cancer, (iv) no previous family history of other tumors, (v) confirmed normal cervical findings through HPV and cytology screening at our hospital, or via cervical biopsy reviewed by two or more pathologists at our institution or through external biopsy review by a panel of two or more pathologists at our hospital. Exclusion criteria were as follows: Samples did not fit the aforementioned inclusion criteria and needed to be excluded, and subjects who were pregnant, lactating or not having sex should also be omitted.

### 2.2 Data collection and sample collection

Information on education level, history of cervical surgery, HPV infection, delivery number, menarche, menopause, dysmenorrhea, history of sex life, and demographic characteristics were obtained for all participants through an on-site questionnaire survey. Some questions were not responded to due to patients’ privacy considerations, and missing values are addressed later in the data preprocessing. Four milliliters of peripheral venous blood were collected from each participant. Genomic DNA (QIAamp DNA Mini Kit (Cat. 51,306, QIAGEN)) was extracted from the hematocrit brown layer of blood samples. The selection of SNPs was based on preliminary experimental results and previously published literature (12). Following target region sequencing, the final set of 59 SNPs was determined using criteria such as a *p*-value of Trend-test <0.1 and the exclusion of linked loci (Supplementary Table S1). To construct the library for sequencing, the genome DNA of each sample was randomly interrupted into fragments (around 250 bp to 300 bp) and adaptors were ligated to both ends of these fragments. After purification, the library was amplified using LM-PCR and hybridized with SureSelect Biotinylated RNA Library (BAITS) for fragment enrichment. These enriched fragments were amplified again by LM-PCR. After the quality check, the DNA library was ultimately sequenced. The DNA library was sequenced using the Illumina HiSeq 2000 platform after the quality test.

Data quality control includes the removal of adaptor-contaminated reads and low-quality reads (defined as N proportion ≥ 10% or ≥ 50% of bases with Q ≤ 5). The accuracy of the clean data should be >90% for Q20 and > 85% for Q30.

### 2.3 Data preprocess

Among the clinical data acquired from the on-site questionnaire, variables with missing data rates of >15% and samples with missing data rates of >50% were removed. We calculated the remaining missing data using the k-nearest neighbor (k-NN) imputation algorithm. SNPs with a mutation rate below 3% or above 97% were excluded. By incorporating the clinical information with SNPs, we acquired the final dataset as 9 clinical factors and 47 mutation variables for 464 samples.

### 2.4 Model building and statistical analysis

**Figure 1:**
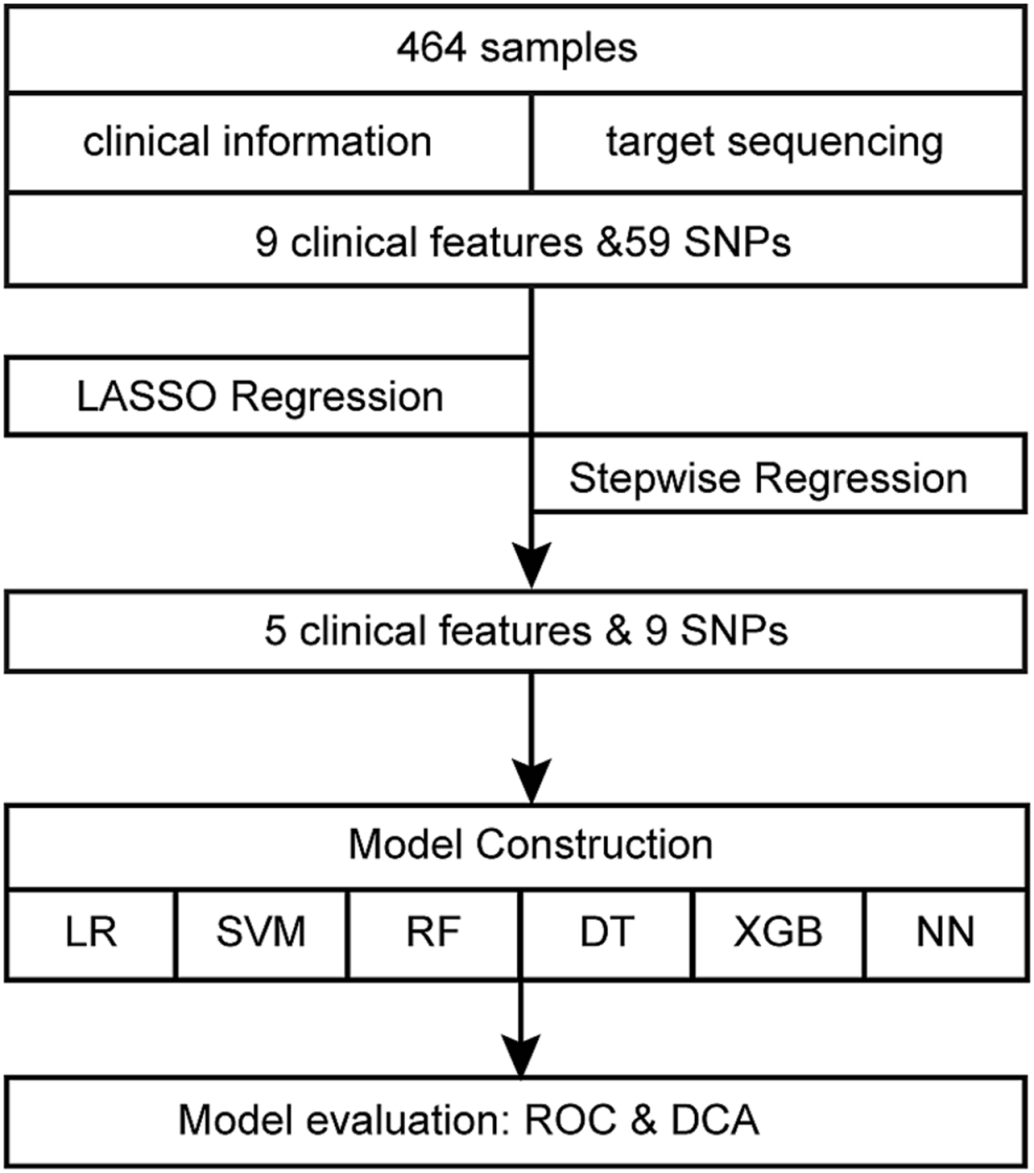
Flow diagram of the whole research.

Figure 1 displays the comprehensive flowchart. After running each variable via univariate logistic regression, the variables with *p*-value <0.05 were selected for the subsequent analysis. The least absolute shrinkage and selection operator (LASSO) regression with ten-fold cross-validation was applied to dimensionality reduction. After further selection through backward stepwise regression, the retained features were modeled by using logistic regression (LR), support vector machines (SVM), random forests (RF), decision trees (DT), eXtreme Gradient Boosting (XGBoost) and neural network (NN). The initial cohort of 464 samples underwent a random split, resulting in two distinct datasets: a training set comprising 325 samples and a test set consisting of 139 samples, with a partition ratio of 7:3. The training set underwent an internal 10-fold cross-validation process, employing nine folds for model building and reserving one fold for validation. Performance metrics for both the training and validation sets were averaged over iterations within the cross-validation. Specifically, nine folds of the training set, were used for training the model, maintaining a sufficient event size (145 events) relative to the 14 variables included, as recommended by the 10-EPV (ten events per variable) guideline (13, 14). The optimal model was selected based on these averaged metrics. Subsequently, the entire training set was utilized for final model training, while the test set served as an independent validation set. The model’s performance was evaluated based on the area under the curve (AUC), accuracy, sensitivity, and specificity. By computing the net benefit of the training and validation cohorts and plotting decision curve analysis (DCA), the clinical utility of the logistic regression model was judged. R software (version 4.1.1) was used for all statistical analyses. R packages “simputation,” “glmnet,” “caret,” “pROC,” “broom,” “forestplot,” “grid,” “magrittr,” “tinytex,” “checkmate,” “rmda,” “e1071,” “randomForest,” “rpart,” “rpart.plot,” “dplyr,” “xgboost,” “tidyverse” and “neuralnet” were used in this investigation. Statistical significance is indicated by bilateral *p*-value <0.05.

## 3 Results

### 3.1 Basic characteristics of the study subjects

The demographic details and clinical features of the population in this study are shown in Table 1. Regarding age, BMI, education level, history of prior cervical surgery, HPV infection, number of births, number of live births, number of transvaginal births, age of menarche, duration of menstruation, and dysmenorrhea, there were no statistically significant differences between the training set and validation set in both patient and control groups (*p*-value >0.05), indicating that the two sets were comparable.

**Table 1:** Demographic and clinical characteristics of patients between training and validation cohorts.

	Controls			Patients		
	Training set	Validation set	p.overall	Training set	Validation set	p.overall
	*N* = 180	*N* = 78		*N* = 145	*N* = 61	
Education level			0.923			0.833
Illiterate	2 (1.14%)	1 (1.32%)		8 (6.30%)	3 (5.45%)	
Primary school	20 (11.4%)	7 (9.21%)		24 (18.9%)	9 (16.4%)	
Junior high school	48 (27.3%)	18 (23.7%)		30 (23.6%)	17 (30.9%)	
High school/Junior college	40 (22.7%)	18 (23.7%)		25 (19.7%)	8 (14.5%)	
College/Vocational college or above	36 (20.5%)	15 (19.7%)		18 (14.2%)	6 (10.9%)	
University and above	30 (17.0%)	17 (22.4%)		22 (17.3%)	12 (21.8%)	
History of cervical surgery			0.249			0.513
No	153 (85.0%)	61 (78.2%)		136 (93.8%)	59 (96.7%)	
Yes	27 (15.0%)	17 (21.8%)		9 (6.21%)	2 (3.28%)	
HPV infection			0.213			0.436
No	56 (32.2%)	31 (41.3%)		6 (5.77%)	1 (2.13%)	
Yes	118 (67.8%)	44 (58.7%)		98 (94.2%)	46 (97.9%)	
Number of birth			1			0.519
NA	1 (0.56%)	0 (0.00%)		0 (0.00%)	0 (0.00%)	
≤2	170 (94.4%)	74 (94.9%)		126 (86.9%)	51 (83.6%)	
≥3	9 (5.00%)	4 (5.13%)		19 (13.1%)	10 (16.4%)	
Number of alivebirth			1			0.519
NA	1 (0.56%)	0 (0.00%)		0 (0.00%)	0 (0.00%)	
≤2	170 (94.4%)	75 (96.2%)		126 (86.9%)	51 (83.6%)	
≥3	9 (5.00%)	3 (3.85%)		19 (13.1%)	10 (16.4%)	
Number of transvaginal births			0.874			0.751
NA	2 (1.11%)	0 (0.00%)		1 (0.69%)	0 (0.00%)	
≤2	171 (95.0%)	74 (94.9%)		127 (87.6%)	52 (85.2%)	
≥3	7 (3.89%)	4 (5.13%)		17 (11.7%)	9 (14.8%)	
Age of menarche^a^	13.5 (1.38)	13.6 (1.80)	0.777	13.8 (1.67)	14.1 (1.58)	0.194
Duration of menstrual			0.941			0.414
NA	19 (10.6%)	8 (10.3%)		7 (4.83%)	6 (9.84%)	
>7	16 (8.89%)	8 (10.3%)		4 (2.76%)	2 (3.28%)	
≤7	145 (80.6%)	62 (79.5%)		134 (92.4%)	53 (86.9%)	
Dysmenorrhea			0.41			0.17
No	111 (68.5%)	44 (62.0%)		103 (74.6%)	50 (84.7%)	
Yes	51 (31.5%)	27 (38.0%)		35 (25.4%)	9 (15.3%)	
AGE^a^	41.8 (10.6)	40.4 (10.2)	0.306	43.0 (10.8)	41.4 (9.64)	0.285
BMI^b^			0.198			0.845
NA	13 (7.22%)	5 (6.41%)		13 (8.97%)	4 (6.56%)	
<18.5	12 (6.67%)	11 (14.1%)		14 (9.66%)	8 (13.1%)	
>24	48 (26.7%)	15 (19.2%)		28 (19.3%)	11 (18.0%)	
18.5–24	107 (59.4%)	47 (60.3%)		90 (62.1%)	38 (62.3%)	

Unless otherwise indicated, values are number of patients with percentage in parentheses.

a Values are the mean with standard deviation in parentheses.

b BMI (body mass index) = weight (kg)/height^2^(m^2^).

### 3.2 Variable selection

**Figure 2:**
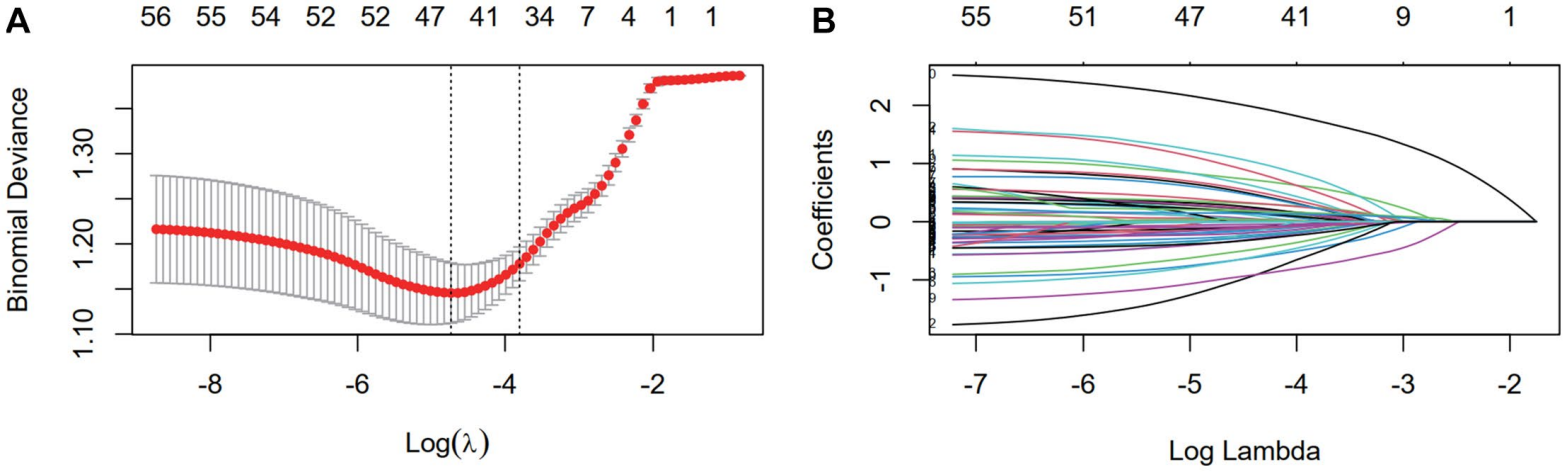
The dimensionality reduction of 9 clinical features and 59 mutation features by LASSO. **(A)** Selection of the tuning parameter (λ) via 10-fold cross-validation based on minimum criteria. Binomial deviances from the LASSO regression cross-validation procedure were plotted as a function of log (λ). The optimal λ value of 0.014 was selected. **(B)** LASSO coefficient profiles of the 68 variables. As the value of λ decreased, the degree of model compression increased and the function of the model to select important variables increased.

Nine clinical variables (education level, history of prior cervical surgery, HPV infection, number of births, number of live births, number of transvaginal births, age of menarche, duration of menstruation, and dysmenorrhea) showed statistically significant differences in the univariate logistic regression analysis (Table 2). After data preprocessing, LASSO regression (Figure 2) was used to filter variables among the 47 mutant loci and the 9 clinical variables indicated above. 15variables (6 clinical variables +9 SNPs) with nonzero coefficients were obtained at lambda = 0.040. Stepwise regression was used to acquire 14 variables (5 clinical factors +9SNPs) to further minimize the variables and boost clinical utility.

**Table 2:** Variate screening using univariate logistic regression.

	Control	Case	p.overall
	*N* = 258	*N* = 206	
**Education level**			0.002
Illiterate	3 (1.16%)	15 (7.28%)	
Primary school	27 (10.5%)	35 (17.0%)	
Junior high school	67 (26.0%)	52 (25.2%)	
High school/Junior college	59 (22.9%)	38 (18.4%)	
College/Vocational college or above	52 (20.2%)	28 (13.6%)	
University and above	50 (19.4%)	38 (18.4%)	
**History of cervical surgery**			<0.001
No	214 (82.9%)	195 (94.7%)	
Yes	44 (17.1%)	11 (5.34%)	
**HPV infection**			<0.001
No	92 (35.7%)	9 (4.37%)	
Yes	166 (64.3%)	197 (95.6%)	
**Number of birth**	1.28 ± 0.82	1.54 ± 1.03	0.003
**Number of alive birth**	1.22 ± 0.81	1.52 ± 1.04	0.001
**Number of transvaginal births**	0.93 ± 0.94	1.27 ± 1.17	0.001
**Age of menarche**	13.6 ± 1.52	13.9 ± 1.65	0.033
**Duration of menstrual**	5.81 ± 1.99	5.31 ± 1.53	0.002
**Dysmenorrhea**			0.028
No	177 (68.6%)	161 (78.2%)	
Yes	81 (31.4%)	45 (21.8%)	

### 3.3 Model construction and validation

Six models were developed using 10-fold cross-validation. Supplementary Table S2 displays the average evaluation metrics, demonstrating that the LR, XGBoost, and NN models outperformed the others.

Based on the 14 variables acquired above, we created a multivariate logistic regression model using the imputation approach, and the Akaike information criterion (AIC) was 363.82. Placing cut points at the maximum “Youden index” (0.288, which placed the best cutoff point with both high sensitivity and specificity), we divided samples into two groups (control, patient) to calculate confusion matrix according to yhat value. SVM has four widely used kernel functions-the linear function, polynomial function, sigmoid function, and radial basis function. By comparing the prediction accuracy of the four kernel function modeling in the e1071 package, the best SVM model—rbf kernel function model—was obtained at the cost of 1 with an accuracy of 0.645.

The error value of the RF model achieves the smallest when the number of decision trees is 16. As the number of decision trees rises, the model’s error steadily declines. The three most crucial factors are HPV, dysmenorrhea, and history of cervical surgery after rating the critical components of the RF model (Supplementary Figure S1).

For the DT model, 0.011 was the ideal cp value. The input variables for the DT model were HPV and history of cervical surgery, and the final number size was 3 based on the matching ordering of significant characteristics. The Supplementary Figure S2 displays the decision tree model and outcomes. In the training dataset, the model successfully categorized 71.8% of the samples.

In comparison, the XGBoost model and the NN model exhibited superior performance, closely trailing the predictive capacity of the LR model, achieving an AUC of 0.80. rs3741378, rs2274933 and dysmenorrhea were assessed as top 3 important variables in XGB model, while HPV, rs148927246 and rs141000672 ranks top 3 in NN model.

**Figure 3:**
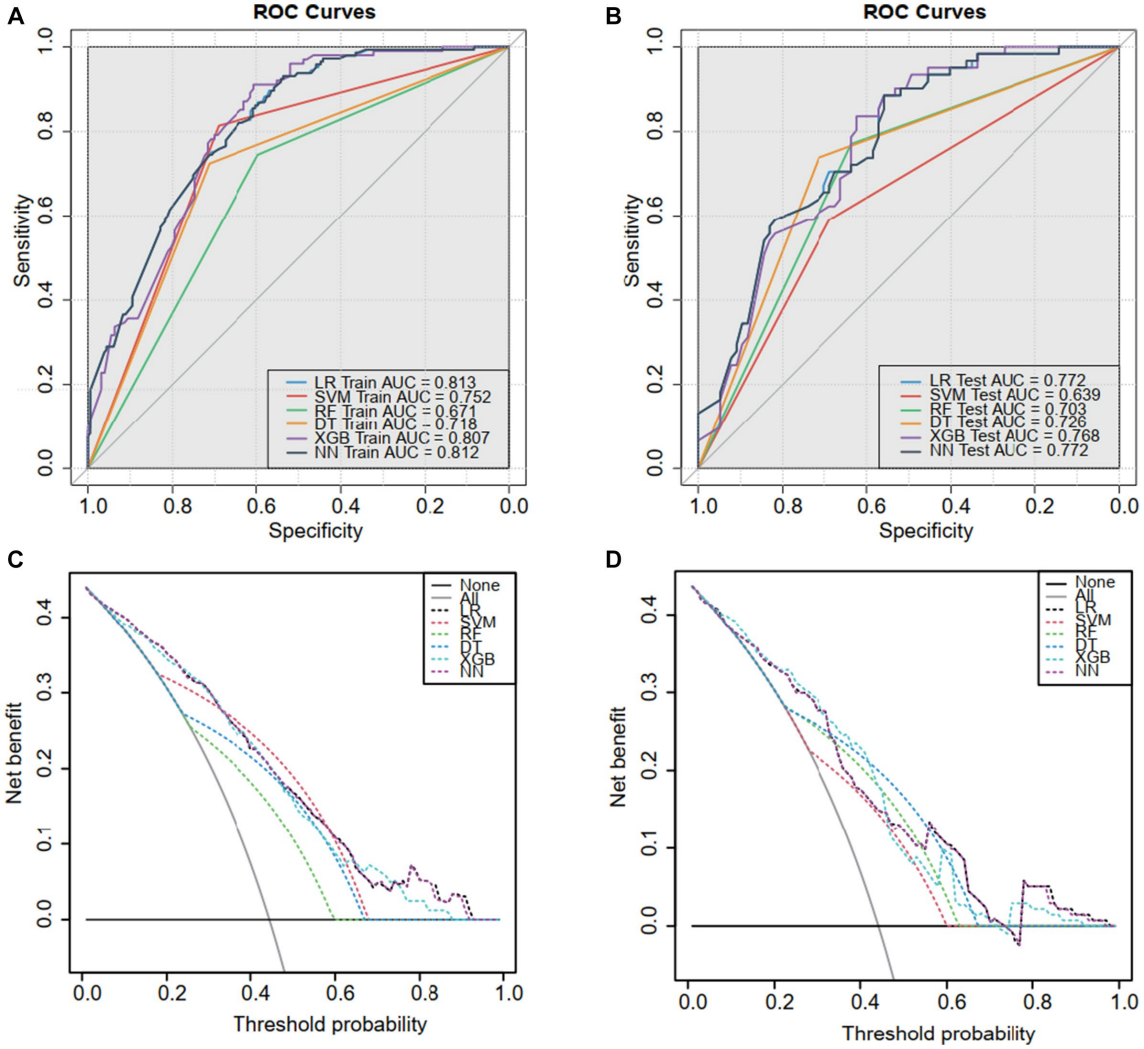
The receiver operating characteristic (ROC) curves and decision curve analysis (DCA). ROC for the **(A)** training sets and **(B)** validation sets. Based on the area under the decision curve, DCA was used to assess the clinical utility of the logistic model. The area of the **(C)** training set and **(D)** validation set is greater than the “treat all” (gray) or “no treatment” (black) strategy. This indicates that the logistic model has good utility in clinical decision making.

The discriminability of the six models in the training and validation sets was assessed using the ROC curve analysis, and the AUCs were established (Figures 3A,B). We chose the logistic regression model as our final model for its relatively high performance, simplicity, and interpretability, making it practical for real-world applications (Table 3).

**Table 3:** Performance of four models for predicting the occurrence of cervical cancer.

Model	AUC		Accuracy		Sensitivity		Specificity	
	Training	Validation	Training	Validation	Training	Validation	Training	Validation
Logistic regression	0.813	0.772	0.712	0.688	0.907	0.870	0.616	0.598
Supportive vector machine	0.752	0.639	0.745	0.645	0.678	0.600	0.822	0.679
Random forest	0.671	0.703	0.663	0.696	0.745	0.778	0.597	0.627
Decisive tree	0.718	0.726	0.718	0.725	0.763	0.775	0.669	0.672
eXtreme gradient boosting	0.807	0.768	0.741	0.703	0.911	0.836	0.606	0.597
Neural network	0.812	0.772	0.712	0.688	0.931	0.902	0.536	0.519

### 3.4 Evaluation of the LR model

**Figure 4:**
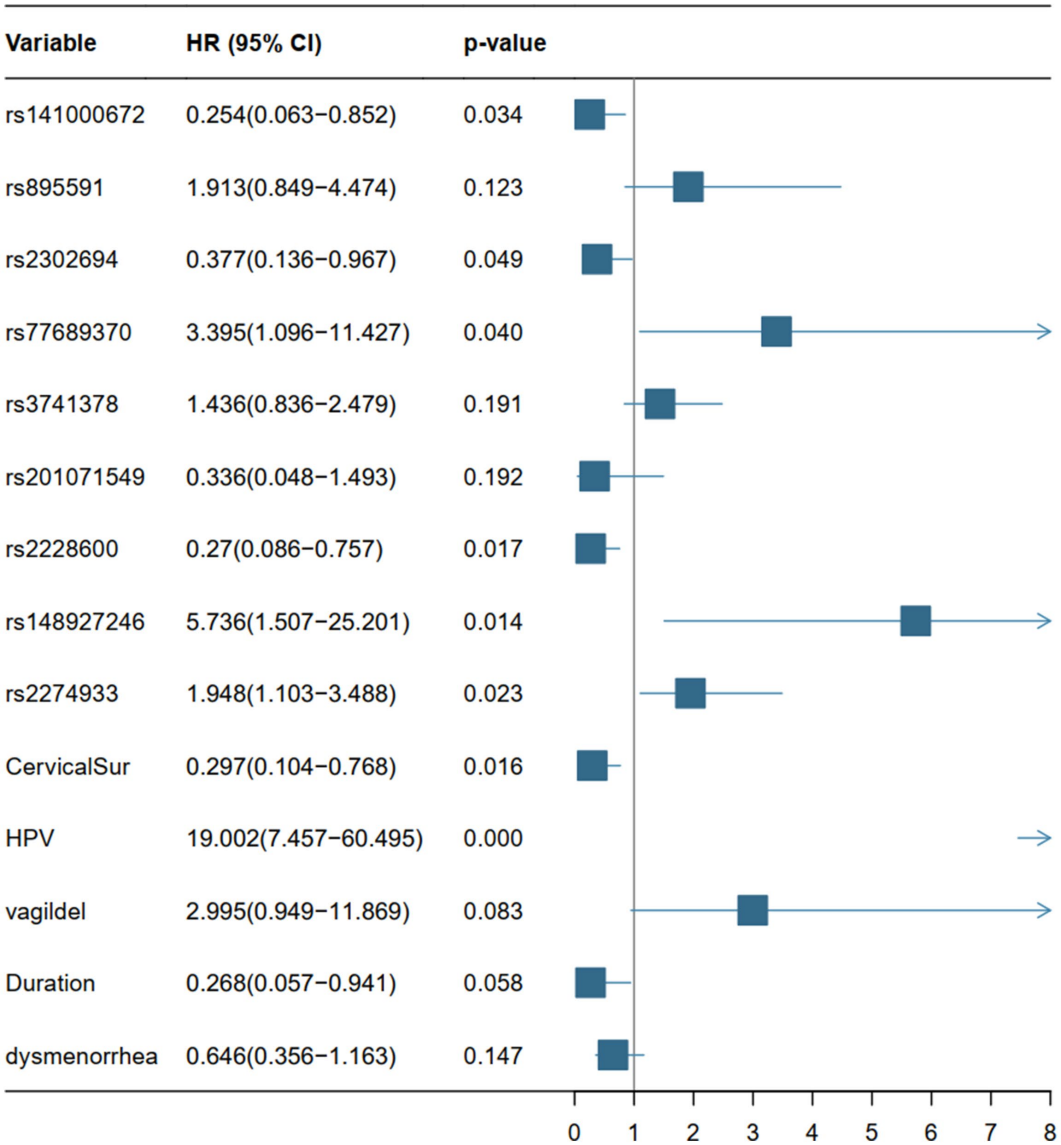
Forest plots based on the *p*-value, HR values (95% CI) of the 14 variables of the LR model. HR, hazard ratio; CI, confidence interval.

The DCA analysis (Figures 3C,D) concluded a good performance of the LR model in terms of clinical applications. The forest plot for the LR model appears in Figure 4, rs141000672, rs2302694, rs77689370, rs2228600, rs148927246, rs2274933, history of cervical surgery, and HPV infection were independently associated with cervical carcinogenesis, where rs141000672, rs2302694, rs2228600, history of cervical surgery were protective factors, and rs77689370, rs148927246, rs2274933 and HPV infection were identified as risk factors.

## 4 Discussion

This research aimed to uncover factors associated with cervical cancer and precancerous lesions, considering both external factors (such as an individual’s education level, menstrual history, and marital status) and internal factors related to genetic susceptibility. We carefully analyzed mutations in 59 specific genetic markers (SNPs) and integrated these findings into our modeling approach. We explored several modeling methods and found that the XGBoost model, combining multiple factors, showed strong predictive abilities, making it our preferred choice for predicting cervical cancer development.

Several clinical diagnostic prediction models for cervical cancer have been developed recently. The study by Van den Helder et al. showcased the application of hrHPV DNA testing and DNA methylation analysis in urine for detecting CIN2, CIN3, and cervical cancer. Their findings, boasting an AUC of 0.84 along with high sensitivity and specificity, provide a promising perspective on detecting cervical cancer and precancerous lesions (15). Furthermore, an advanced Stacking-Integrated Machine Learning (SIML) model was developed to identify high-risk individuals for cervical cancer. This model achieved an AUC of 0.877, with a sensitivity of 81.8% and specificity of 81.9%, demonstrating its potential for accurate risk assessment based on demographic, behavioral, and clinical factors (16). Fu et al. asserted that a colposcopy-based multi-image deep learning model that incorporates the results of both an HPV test and a cytology test would produce results with higher sensitivity and specificity than the cytology-HPV diagnostic model or the colposcopy-based multi-image deep learning model applied independently (17). Another study successfully stratifies high-grade cervical lesions employing sequencing and machine learning as a valuable addition to the current comprehensive triage method (18). However, none of the aforementioned studies highlighted the integration of germline mutations detected through SNP analysis as a pivotal aspect for the early warning of cervical cancer, which is the distinguishing feature of our model. Our model uniquely incorporates germline mutations detected through SNP analysis, highlighting the novel inclusion of genetic susceptibility factors for early detection of cervical cancer.

According to previous research, HPV is unquestionably the most significant risk factor for cervical carcinogenesis. Cervical precancerous and invasive carcinoma need ongoing high-risk HPV infection of cervical basal epithelial cells with the capacity to divide and differentiate as well as integration of viral DNA with the host genome (19, 20). As a result, high-risk HPV screening is especially relevant as a primary screening method for cervical cancer. The LR model considers a prior history of cervical surgery protective, which makes sense, given that this operation eliminates the anatomical components most likely to develop cervical cancer.

In the LR model, 6 SNPs in 5 genes were independently associated with cervical carcinogenesis, with each SNP exerting its slight effect. We found formerly published evidence on several of these SNPs associated with cancer. HSPG2 (rs141000672) encodes the perlecan protein, and SNPs of HSPG2 (rs12034979, rs6697265, rs6680566, and rs878949) had previously been identified as potential risk factors for the advancement of cervical lesions caused by HPV types 16, 18, and 52 infections (21). In our model, HSPG2 is thought to be a protective factor against cervical cancer. Multiple studies have shown that small structural differences in HSPG2 across diseases have antagonistic effects on tumor formation and metastasis; intact perlecan promotes the development of a vascular supply that supports tumor cell proliferation and the development of a variety of cancers, whereas bioactive perlecan fragments inhibit tumor development by targeting its vascular supply (22, 23). LRP2 (rs2302694) mutations were detected in a range of cancers, with melanoma (28.18%), uterine sarcoma (17.99%), and lung squamous cell carcinoma (16.32%) with the highest mutation rates (24). A pan-cancer study found that LRP2 mutations were linked with increased immune cell infiltration, immune checkpoint gene expression, and significant enrichment of immune-related signaling pathways, as well as a better prognosis, compared to individuals who did not have LRP2 mutations (24). Depletion of Lama5 (rs148927246, rs2274933) in lymph node stromal cells controls immunological responses to T cell migration and function, encourages branching angiogenesis, and modifies Notch signaling, which facilitates colorectal cancer spread to the liver (25, 26). This raises the possibility that it could impact cervix cellular immunity and angiogenesis and encourages cervical carcinogenesis. HPV infection type is connected with polymorphisms in HLA-DRB1 (rs77689370), which ultimately affects how quickly high-risk HPV-infected cervical lesions develop into invasive cervical cancer (27). The clockican family member NCAN (rs2228600) is primarily expressed in neural tissue. The carcinogenesis and malignancy of neuroblastoma (NB) are influenced by NCAN, which also promotes the growth and invasion of glioma cells (28, 29).

Among the six warning models, the decision tree model is clinician-friendly and has high clinical tractability for making decisions from root to leaf nodes, while it exhibited instability in generalization. NN showed competitive performance but slightly lower accuracy compared to LR. On the other hand, the diagnostic performance of the RF, SVM and XGB models was mediocre, and their “black box” characteristics limited clinical interpretability marginally. Relatively, the multivariate LR model is more interpretable and can assist physicians in anticipating the occurrence of cervical cancer. The 8 variants in the LR model were proven to be risk factors of statistical significance, including 2 clinical features and 6 SNPs. Advanced techniques like XGBoost and neural networks demonstrated superior predictive capabilities. Their outperformance in accuracy and AUC proves their ability to handle complex relationships in data, making them promising models that balance interpretability and performance.

All in all, our model is positioned as a valuable complement to established screening methods like the ThinPrep Cytology Test (TCT) and HPV testing, especially beneficial for individuals exhibiting positive HPV results alongside normal TCT findings. The potential lies in enhancing both sensitivity and specificity in clinical screening, facilitating the identification of high-risk individuals who might otherwise be overlooked. To ascertain its precise application in clinical practice, rigorous empirical research and thorough clinical validation are imperative. Despite these promising aspects, the present study has certain limitations. Primarily, the retrospective collection of clinical variables through self-reported data in a case–control setup may introduce measurement errors and recall biases, potentially impacting the predictive accuracy of the data. Additionally, the study’s limited sample size significantly reduces statistical power within both the training and validation cohorts. Despite this limitation, we have endeavored to emphasize the robustness of our internal validation procedures, including the use of 10-fold cross-validation, to mitigate potential biases and enhance the reliability of our findings within the scope of available resources.

In summary, our model incorporating both clinical features and SNPs contributes valuable insights toward predicting cervical carcinoma. While showing promising predictive ability, further refinement and validation are essential to ascertain its full clinical utility.

## Data availability statement

The variation data reported in this paper have been deposited in the Genome Variation Map (GVM) in the National Genomics Data Center, Beijing Institute of Genomics, Chinese Academy of Sciences and China National Center for Bioinformation (https://bigd.big.ac.cn/gvm), under accession number GVM000690.

## Ethics statement

The studies involving humans were approved by the Ethics Committee of Tongji Hospital, Tongji Medical College, Huazhong University of Science and Technology. The studies were conducted in accordance with the local legislation and institutional requirements. The participants provided their written informed consent to participate in this study.

## Author contributions

XL: Data curation, Formal analysis, Methodology, Software, Validation, Writing – original draft. RN: Data curation, Methodology, Conceptualization, Investigation, Supervision, Writing – review & editing. BX: Data curation, Methodology, Writing – original draft. SM: Methodology, Formal analysis, Writing – review & editing. HS: Writing – review & editing, Investigation, Supervision. XF: Data curation, Formal analysis, Methodology, Writing – original draft. SL: Conceptualization, Funding acquisition, Investigation, Project administration, Supervision, Writing – review & editing.
