# Supplementary figures and images for: A multi-variable predictive warning model for cervical cancer using clinical and SNPs data
Source: Front Med (Lausanne). 2024 Feb 22;11:1294230. doi: 10.3389/fmed.2024.1294230 (PMC10918689; doi:10.3389/fmed.2024.1294230)

## rf\_model\_adj

HPV  
CervicalSur  
ALIVEBIRTH  
rs2274933  
Duration  
rs2302694  
rs3741378  
rs148927246  
rs895591

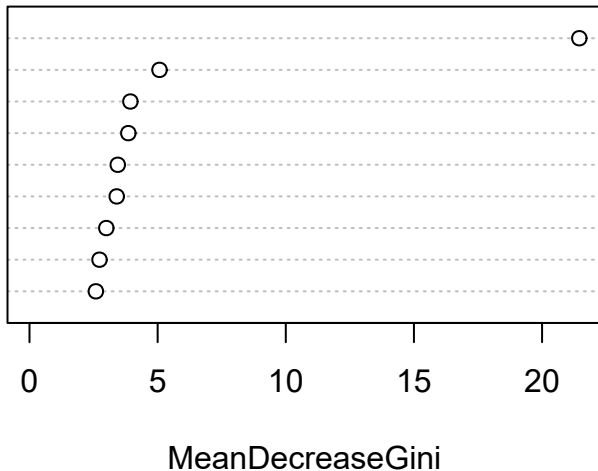

Supplement: Supplementary file 1 [file Data_Sheet_1.PDF]

# Decision Tree

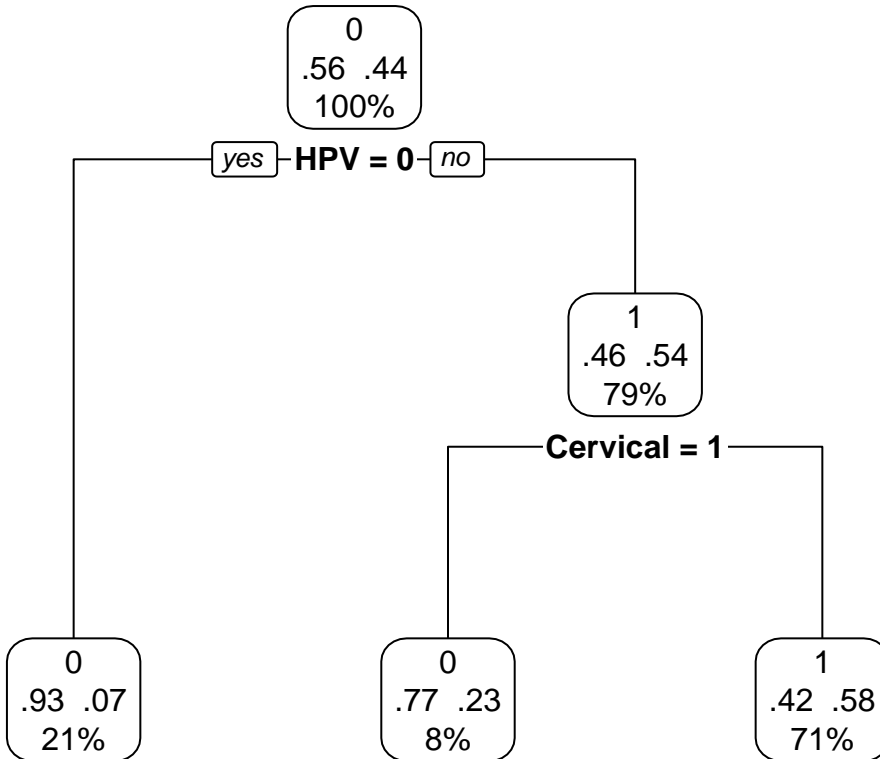

Supplement: Supplementary file 2 [file Data_Sheet_2.PDF]
